# Las ciencias básicas odontológicas como fundamentos para la práctica clínica

**DOI:** 10.21142/2523-2754-0902-2021-053

**Published:** 2021-06-21

**Authors:** Angela Quispe-Salcedo

**Affiliations:** 1 Carrera de Estomatología, Facultad de Ciencias de la Salud de la Universidad Científica del Sur. Lima, Perú. aquispesa@cientifica.edu.pe, aquispesa@dent.niigata-u.ac.jp Universidad Científica del Sur Carrera de Estomatología Facultad de Ciencias de la Salud Universidad Científica del Sur Lima Peru aquispesa@cientifica.edu.pe aquispesa@dent.niigata-u.ac.jp; 2 Division of Anatomy and Cell Biology of the Hard Tissues, Niigata University Graduate School of Medical and Dental Sciences. Niigata, Japan. Division of Anatomy and Cell Biology of the Hard Tissues Niigata University Graduate School of Medical and Dental Sciences Niigata Japan

Las carreras del área de ciencias de la salud combinan la adquisición de conocimientos básicos y habilidades específicas para el desempeño clínico desde etapas tempranas de la formación profesional. En el caso de la odontología, estas habilidades son sumamente complejas, lo cual implica que el estudiante posea un adecuado conocimiento previo de las estructuras de la cavidad oral y su fisiología. 

Por tal motivo, los primeros semestres de la currícula de las escuelas dentales a nivel mundial centran sus esfuerzos en brindar una intensiva formación en ciencias básicas generales y aplicadas, a través de cursos como anatomía humana, biofísica, microbiología, embriología e histología, fisiología, entre otros ^1^^,^^2^. El objetivo de integrar estos cursos en la malla curricular es obtener un vínculo sólido entre las bases biológicas y los procedimientos clínicos que serán introducidos paulatinamente en los semestres siguientes. De esta manera, el alumno se aleja de la visión tecnicista de la carrera al entender la importancia de cada paso a seguir en la recuperación de las estructuras afectadas por las enfermedades orales ^3^.

Lamentablemente, la experiencia educativa nos demuestra lo inestable de esta vinculación. Cuando los cursos básicos no están adecuadamente estructurados o son dictados por personas sin experiencia o formación en el área, la vinculación básica-clínica es deficiente. El estudiante no logra entender la importancia de los procedimientos clínicos y sus consecuencias a largo plazo, y eso se hace evidente en la calidad profesional de los egresados, lo que pone en riesgo la correcta aplicabilidad de criterios odontológicos para la toma de decisiones en el tratamiento del paciente.

Una plana docente motivada y con experiencia en sus respectivas áreas del conocimiento básico, y una correcta organización de la malla curricular son sumamente importantes para que el alumno pueda establecer ese vínculo fundamental que determinará su autoconfianza y seguridad en su práctica clínica. Adicionalmente a la formación básica de los primeros semestres, resultaría ideal que los docentes de los cursos básicos puedan formar parte de las cátedras avanzadas en las que se aplican situaciones de “aprendizaje basado en problemas”, que toma escenarios clínicos de diversa índole; de esta manera, el alumno puede recordar los conocimientos previos con la guía de quienes lo instruyeron en ese momento ^4^. 

Finalmente, es fundamental que las escuelas o facultades de odontología de nuestro país puedan incentivar las actividades de investigación en la plana docente de los cursos básicos odontológicos, y desarrollen diversas líneas de trabajo por área. Motivemos a nuestros alumnos a buscar siempre la excelencia y profundizar sus conocimientos básicos más allá de los contenidos del sílabo. Recordemos que todo procedimiento, antes de ser trasladado a la clínica, fue primero un hallazgo científico que surgió del esfuerzo y la dedicación de muchos dentistas-científicos, a través de la investigación básica odontológica.
